# Low Vision Rehabilitation Referral Characteristics for Patients with Neovascular Age-Related Macular Degeneration

**DOI:** 10.3390/healthcare13010064

**Published:** 2025-01-01

**Authors:** Simon D. Archambault, Courtney Sweeny, Mahesh Bhardwaj, David J. Ramsey

**Affiliations:** 1 Division of Ophthalmology, Department of Surgery, UMass Chan—Lahey School of Medicine, Burlington, MA 01805, USA; 2 Department of Ophthalmology, Tufts University School of Medicine, Boston, MA 02111, USA; 3 Department of Graduate Studies, New England College of Optometry, Boston, MA 02115, USA

**Keywords:** age-related macular degeneration, electronic health record, low vision, delivery of healthcare, quality improvement

## Abstract

**Background**: Despite evidence that low vision rehabilitation (LVR) services can improve visual function in patients with neovascular age-related macular degeneration (nAMD), many patients are not directed to access these resources. This study was conducted to determine factors associated with LVR referral and to assess the visual outcomes from completed evaluations. **Methods**: The study comprised a retrospective, cross-sectional analysis of patients with nAMD. Referrals for LVR services were extracted from the electronic health record (EHR). The effectiveness of each evaluation was determined by assessing the change in best corrected visual acuity (BCVA) achieved after distance refraction. Costs, quality-adjusted life years (QALYs), and incremental costs per-QALY-gained were calculated based upon the better-seeing eye by using a willingness-to-pay threshold of $50,000/QALY. **Results**: Out of 560 eligible patients with nAMD, 110 were referred for LVR (19.6%). Referral was more common for individuals who qualified as having low vision, based upon the visual acuity of the better-seeing eye (adjusted odds ratio [aOR], 3.214; 95% confidence interval [CI], 1.920–5.380, *p* < 0.001), had bilateral nAMD (aOR, 1.592; 95% CI, 1.017–2.492, *p* = 0.042), or had commercial health insurance compared to those who had Medicare (aOR, 2.887; 95% CI, 1.041–8.009, *p* = 0.042). Most patients referred completed LVR appointments (86%). More than half of the patients achieved improved BCVA for their better-seeing eye (53%) yielding an average gain of 0.04 QALYs/patient at a cost of $3504/QALY. The estimated net monetary benefit was $1704 per evaluation completed. **Conclusions**: Most patients with nAMD achieved improvements in visual function after low vision evaluation, yielding improvements in vision-related quality of life at a reasonable cost.

## 1. Introduction

One out of every ten Americans above the age of eighty experience permanent visual impairment resulting from advanced age-related macular degeneration (AMD) [1,2]. This number is expected to rise significantly over the next twenty-five years as the United States population rapidly continues to age [1]. Visual impairment ranks among the top ten causes of disability in the United States [2] and is associated with difficulties performing instrumental activities of daily living [3], lower quality of life [3,4], elevated rates of depression [3,5], and increased morbidity and mortality [6]. Visual impairment additionally takes a significant toll on the United States economy. As of 2020, the estimated global annual economic loss in productivity associated with visual impairment in the United States surpassed $400 billion [7].

While neovascular age-related macular degeneration (nAMD) accounts for only 15% of advanced AMD cases, it is responsible for over 80% of all vision loss secondary to the disease [1,2,8]. Treatment for nAMD with anti-vascular endothelial growth factor (anti-VEGF) agents tend to slow vision loss and, in some cases, patients may experience short-term visual gains [9]. However, despite ongoing treatment, the natural course of the disease results in decreased visual acuity, contrast sensitivity, and vision-related quality of life [10]. Low vision rehabilitation (LVR) can help visually impaired individuals optimize their remaining vision and improve their independence [11]. LVR can be of particular benefit to those with advanced macular diseases, as demonstrated in the Veterans Affairs Low Vision Intervention Trial [12]. Despite evidence that LVR can further improve visual function, many patients with nAMD do not receive a referral to these services. Lack of patient motivation, provider awareness, clear referral pathways, sufficient time during clinic visits, and poor coordination of care have all been identified as impediments to LVR referral [13]. While previous studies have examined provider referral patterns in patients with intermediate age-related macular degeneration [14] there is little literature examining referral patterns in patients with more advanced disease.

The aim of this study was to evaluate the rate of LVR referrals and identify patient factors associated with LVR referrals for patients with nAMD. After identifying factors associated with referrals, we evaluated visual outcomes and aspects related to the cost-effectiveness of LVR in this population. We also analyzed potential benefits of vision-related quality of life, such as economic considerations associated with the cost of these interventions.

## 2. Materials and Methods

The present study was completed as part of a quality improvement project at Lahey Hospital & Medical Center (Burlington, MA, USA) to ascertain the characteristics associated with referral and utilization of LVR services for patients with nAMD at a large academic ophthalmology practice. The investigation was conducted in compliance with the tenets of the Declaration of Helsinki and received Research Ethics Board approval. Information was gathered and secured in compliance with the Health Insurance Portability and Accountability Act.

### 2.1. Study Participants

A retrospective, cross-sectional analysis was conducted to identify patients diagnosed with nAMD in the 12-month period 1 July 2021 to 30 June 2022. Criteria for LVR referral, as defined by the American Academy of Ophthalmology (AAO), included having a best-corrected visual acuity (BCVA) of worse than 20/40 in the better eye, a central scotoma, a visual field of less than 10 degrees around central fixation, or loss of contrast sensitivity [15]. Patients whose BCVA for the better-seeing eye was between 20/40 and 20/70, inclusive of these endpoints, were classified as having “mild” visual impairment, whereas those with a BCVA below 20/70 were classified as having low vision. Similarly, legal blindness was defined as having a BCVA worse than 20/400 in the better-seeing eye or a visual field of less than 10 degrees around central fixation. Finally, the subset of patients with BCVA for the worse-seeing eye equal to or worse than to 20/200 with a better-seeing eye with BCVA equal to or better than 20/70 were considered functionally monocular. All definitions aligned with the categories established by the 2019 World Health Organization (WHO) report on vision [16].

Patients with other confounding retinal conditions (e.g., central serous retinopathy, diabetic retinopathy, myopic degeneration, or retinal vein occlusions) were excluded from the study. Patients who had previously received LVR services were also excluded.

### 2.2. Patient Characteristics

Patient characteristics were compared between those who received a referral to LVR and those for whom a referral was not documented. Demographic data (age, sex, race/ethnicity, primary language spoken, type of health insurance, employment status, estimated mean household income, distance from the eye clinic) was extracted from the data available in the electronic health record (EHR) by means of a customized reporting tool (EPIC systems Inc., Verona, WI, USA) [17]. Insurance type was classified as either commercial insurance (insurance offered by a private-sector insurance company), Medicare (a federal U.S. insurance program for individuals 65 years or older and certain younger individuals), Medicaid (federal United States insurance program offered to people with limited income or resources) or other governmental insurance (non-Medicaid or Medicare insurance offered by a federal or state government). An Excel VBA program was used to access Microsoft Maps, which calculated the number of miles between each patient’s home and the clinic by zip code [17]. Mean household income was approximated by dividing the total income for a patient’s zip code based upon the 2018 U.S. Census Data by the number of returns for that zip code, as previously described [18]. Clinical characteristics, including nAMD severity, visual acuity, and visual impairment status, were documented for every patient. The extent of visual impairment was defined as (1) low vision, (2) legal blindness, or (3) monocular status as defined by the World Health Organization’s Study Group on the Prevention of Blindness [16].

### 2.3. Low Vision Rehabilitation Utilization

A patient was considered to have received LVR services if they completed a clinic visit within the study period. Because of variations in documentation, as well as the type and extent of services provided, LVR effectiveness was quantified by comparing the presenting visual acuity with the BCVA after distance refraction by a low vision specialist. Costs, quality-adjusted life years (QALYs), and incremental costs per QALY gained through utilization of LVR services were analyzed. Finally, the eyecare providers of patients who had low vision but were not documented to have had a referral for LVR services within the study period were notified and asked to consider placing an order for a consultation, if appropriate.

### 2.4. Statistical Analysis

Data were encoded and analyzed using SPSS^®^ Statistics version 28.0 (IBM Corp, Armonk, New York, NY, USA). Snellen visual acuity was converted to the logarithm of the minimum angle of resolution (logMAR) for comparison. Data are presented as mean (±SD) for continuous variables, frequency (count), and relative frequency (percentage) for categorical data. Student’s *t*-test was used to compare normally distributed quantitative variables. For non-normally distributed variables, the Mann–Whitney *U* test was utilized, and results are presented as median with interquartile range (IQR). Change in BCVA after low vision refraction was assessed by using the Wilcoxon Signed-Rank Test. Incremental costs per quality-adjusted life year (QALY) gained were calculated using a willingness-to-pay threshold of $50,000/QALY [19]. The estimated cost for LVR was set at the Medicare professional payment rate for an established patient comprehensive eye examination in 2022 (CPT 92014, $128.39) [20]. The net monetary benefit (NMB) was calculated by using the formula NMB = QALY gained × WTP—Cost [19]. Logistic regression was employed to identify factors associated with referral for LVR services. All tests were 2-sided and *p*-values below 0.05 were regarded as statistically significant.

## 3. Results

### 3.1. Patient Characteristics

In total, 560 patients with nAMD met the inclusion criteria. The study population consisted predominantly of older individuals (median age 84 years [IQR 78 to 89 years]), who identified as White, non-Hispanic (94.3%), were female (62.3%), and were insured by Medicare (94.5%). See Table 1.

Overall, 110 of these patients (19.6%) were referred for LVR services. Patients referred tended to be older (median age 86 years [IQR 80 to 91 years] versus median age 83 years [IQR 78 to 88 years], *p* = 0.020), have other forms of government-sponsored health insurance (2.7% versus 0.4%, *p* = 0.046), and have bilateral nAMD (60.9% versus 45.1%, *p* = 0.003). Of note, none of the small number of patients who had Medicaid health insurance (seven patients, 1.3%) or who lacked health insurance coverage (one patient, 0.2%) were referred for LVR services.

Visual acuity was one of the most significant factors associated with referral for LVR services. Patients referred tended to have worse visual acuity in both the better-seeing (LogMAR 0.514 ± 0.408 versus LogMAR 0.332 ± 0.334, *p* < 0.001) and worse-seeing eye (LogMAR 1.241 ± 0.910 versus LogMAR 0.910 ± 0.796, *p* < 0.001). Patients were also more likely to be referred if the visual acuity in their better-seeing eye was 20/40 or worse (55.5% versus 31.6%, *p* < 0.001), if they had more advanced visual impairment as defined by the World Health Organization (low vision: 31.8% versus 11.8%, *p* < 0.001; legal blindness: 13.6% versus 5.3%, *p* = 0.002), or if they had monocular status (39.1% versus 27.8%, *p* = 0.020). Finally, there was no difference in the rate of referral for LVR services for those patients who were receiving treatment with intravitreal injections compared with observation. However, for the subset of patients who were actively receiving treatment for nAMD, less frequent intravitreal injections were associated with an increased referral rate (average injection interval 6.6 ± 4.2 weeks versus 5.9 ± 4.1 weeks, *p* = 0.044).

Stepwise logistic regression was used to assess the interaction of factors associated with referral to LVR among patients with nAMD. Qualifying as having low vision, based upon the visual acuity of the better-seeing eye (adjusted odds ratio [aOR], 3.214; 95% confidence interval [CI], 1.920–5.380, *p* < 0.001), bilateral nAMD (aOR, 1.592; 95% CI, 1.017–2.492, *p* = 0.042), and commercial insurance (aOR, 2.887; 95% CI, 1.041–8.009, *p* = 0.042) were all included in this model and remained significant stepwise predictors of referral to LVR services (Table 2). The other demographic and clinical variables identified in Table 1 were excluded from our logistic regression model because they lacked unique predictive value with respect to the likelihood of a patient being referred for LVR services.

### 3.2. Low Vision Rehabilitation Utilization

Eighty-six percent of patients who were referred to LVR completed an LVR encounter. Fifty-three percent of those referred had improved BCVA in their better-seeing eye (median ΔBCVA = −0.2 logMAR, IQR = −0.324 to −0.12, *p* < 0.001), with 36% of patients gaining ≥ 2-lines of BCVA. Just over half of patients with low vision who completed an LVR visit (14 of 27 patients) ceased to qualify as having low vision after refraction (*W* = 28, *Z* = −3.04, *p* = 0.002). The improvement in BCVA yielded an average gain of 0.04 QALYs/patient at a cost of $3504/QALY. The estimated net monetary benefit was $1704 per evaluation completed. Finally, each of the eyecare providers for the 53 patients (11.8%) who had low vision but were not documented to have had a referral for LVR services within the study period were notified through the EHR, asking them to consider placing an order for a future consultation, if appropriate.

## 4. Discussion

As the population of the United States and the world ages, the prevalence of AMD continues to rise proportionally [1,8]. The present study, conducted as part of a quality improvement project at a large academic medical center, examined LVR referral patterns for patients with nAMD. In our study, fewer than one in five patients (19.6%) received a referral for LVR services—a similar rate to that observed in previous LVR studies [14]. Patients were more likely to receive a LVR referral if they were older, had other forms of government-sponsored health insurance, bilateral nAMD, or had poor visual acuity in both their better- and worse-seeing eye. Unsurprisingly, patients were also more likely to receive a referral if they met criteria for having legal blindness or monocular status. Many of these same factors have been shown to be associated with low vision referrals for patients with intermediate-stage AMD [14], as well as other ocular diseases [21]. The aim of our study was to evaluate the utilization of LVR services within our practice. Future studies are needed to determine if these efforts, including educational notifications sent out to eyecare providers who had patients with low vision yet to be referred, improved access to these services.

The initiative of making an LVR referral is a duty shared by both optometrists and ophthalmologists. In our study, we did not seek to examine the characteristics of retina providers who made the referrals. However, a large study examining referral patterns to glaucoma specialists found higher referral rates among providers with a high number of patients who had already received LVR services [21]. Additionally, increased referral rates were observed for providers who reported adherence to the American Academy of Ophthalmology’s Vision Rehabilitation Preferred Practice Pattern^®^ recommendations for low vision [22], although only 22% of glaucoma specialists in that study reported following these guidelines [21].

Of the patients in our health system who received a referral, most followed through and completed a consultation. While the rate in our study (86%) is higher than that previously reported (43%) [23], our study population had advanced-stage disease. In one large retrospective analysis of more than 7000 patients, disease severity was correlated with completing a LVR visit [24]. Studies demonstrated that patients are more likely to follow through with an LVR appointment if the service is provided at the same practice where the referral was made [23,24]. Our ophthalmic practice provides LVR services at two hospital-based locations where retinal services are also provided. This may explain the high rate of appointment completion. Future studies should survey the subset of patients who failed to make appointments or access LVR services to better understand the barriers patients with AMD face in obtaining visual rehabilitation.

There are several physician-related factors commonly cited to explain why an eligible patient is not referred. These factors include time constraints imposed at an ophthalmology visit, psychological implications of the referral, and poor provider awareness of services provided by LVR [25]. By contrast, the number of years in practice, practice location or type, and patient volume have not been associated with referral rates [25]. Physicians should be vigilant for patients who meet the definition of low vision based upon the Vision Rehabilitation Preferred Practice Pattern^®^ published by the AAO [22]. While BCVA is the most widely used metric to define low vision status, greater efforts are needed to screen for and measure deficits in contrast sensitivity. Doing so would likely increase the number of patients identified who could benefit from LVR and serve as another important marker of LV status. Additionally, while many scheduling and practice-based factors are out of a clinician’s control, physicians should stay informed about the benefits of LVR services. Such consultations may encompass an assessment of visual and functional needs through a multidisciplinary approach. Vision rehabilitation is also not limited to optometrists and ophthalmologists, but may include occupational and physical therapists, nurses, behavior psychologists, and social workers. An older prospective observational study examined the benefits of LVR services on patients with non-neovascular AMD. The study demonstrated that the utilization of advanced optical aids, along with education on how to properly utilize those aids, led to an increase in the number of patients able to read television subtitles from 7% to 58% and an increase in the number of individuals able to read newspaper text from 0.8% to 92% [26].

Visual impairment has also been associated with increased rates of depression [27] and lower quality of life [3,28]. The 25-item National Eye Institute Visual Function Questionnaire (NEI VFQ-25) was developed to assess vision-specific health-related quality of life in low vision patients, including those with AMD [29]. Patients with advanced AMD score poorly on this instrument, especially on the Vision Subscales (General Vision, Difficulty with Distance Tasks, Difficulty with Near Tasks) and Vision-specific Subscales (Dependency, Role Difficulties, Mental Health, and Social Function) [30]. Importantly, access to LVR services has been associated with increases in both patient quality of life [31] and NEI-VFQ subscale scores (General Vision, Near Activities, Distance Activities, and Peripheral Vision) [32,33]. Our study demonstrated that the completion of an LVR evaluation often resulted in significant improvements in BCVA. This conservatively yielded an estimated net monetary benefit of $1704 per evaluation completed.

By contrast, retina appointments, which often include treatment with intravitreal injections every four or more weeks, can be significantly more costly depending on the type of intravitreal injection administered and the number of injections needed. While off-label bevacizumab (Genentech Inc., San Francisco, CA, USA) costs around $68 for the drug, increasing numbers of patients are being treated with aflibercept ($1644 for the drug) or faricimab-svoa ($2055 for the drug), especially in treatment refractory cases [34]. One large study that examined the experience of more than 170,000 patients found the costs of maintenance injections totaled between $10,702 and $11,351 annually [35]. Furthermore, the primary goal of treatment for such patients is to slow progression of disease, but it typically does not restore vision that has already been lost. By contrast, the frequency of low vision assessments varies, but they are typically conducted about once per year and at a fraction of this cost. Those factors make LVR services one of the most cost-efficient means for adding to a patient’s vision-associated quality of life.

The present study has several limitations. The population examined was derived from a large suburban, academic practice where most patients were older, identified as White, and were insured largely by Medicare. Medicare is a federal health insurance program in the United States administered by the Centers for Medicare and Medicaid Services. It primarily provides coverage for individuals aged 65 and older, as well as younger individuals with qualifying disabilities or specific medical conditions (e.g., end-stage renal disease). However, not all individuals in the U.S., including those over the age of 65, qualify for Medicare. In our study, we relied on primary insurance information documented in the electronic health record (EHR) and did not have access to data to allow for further stratification of insurance types. The high proportion of patients with Medicare in our study likely reflects both the age of participants with AMD-associated vision loss and the well-insured, suburban population in the northeastern U.S. Consequently, our study was not well suited to make full assessment of the influence of insurance status on LVR referrals, because the model presented could not estimate meaningful coefficients for the effect of having Medicaid health insurance or being uninsured, owing to the small sample size of each category. Studies have demonstrated that the type of health insurance can shape vision-related health outcomes and is linked to other social determinants of health [33,36]. Future studies should be performed in larger, more diverse patient populations with a wider range of socioeconomic backgrounds to enhance our understanding of the impact of these factors. The retrospective nature of our study may also introduce a selection bias, whereby eyecare providers may have been more prone to document conversations about LVR services for patients who expressed a willingness to accept a referral. Finally, LVR encompasses a wide range of services and healthcare professionals. We focused on the improvement in BCVA after low vision refraction as our primary outcome measure because of its uniform documentation in the EHR. Many of the other benefits provided by LVR consultations are not as easy to quantify or are unevenly documented. Future prospective studies should use evidence-based quantitative surveys such as the NEI-VFQ-25 to better examine vision-specific health-related quality of life in low vision patients [29].

## 5. Conclusions

In conclusion, our study demonstrated that many patients with nAMD experienced a significant improvement in BCVA after completion of an LVR evaluation. Many patients with nAMD are considered to have low vision and could therefore benefit from LVR services. Patients with low vision or bilateral nAMD were more likely to be referred by retina providers, and most patients who received an LVR referral followed through with those consultations. Despite the proven benefits of LVR in enhancing vision-related quality of life, these services remain underutilized, even though they are affordable from a population cost perspective. Further efforts are needed to improve the rate at which patients with nAMD access LVR services.

## Figures and Tables

**Table 1:** Demographic and Clinical Characteristics of Patients with nAMD.

		Group			
Characteristic	All Patients (n = 560)	Referred (n = 110)	Not Referred (n = 450)	Difference, % (95% CI)	*p* ^†^
**Age, Years**					
Median (IQR)	84 (78 to 89)	86 (80 to 91)	83 (78 to 88)	-	**0.020**
**Sex, % (n)**					
Female	62.3% (349)	64.5% (71)	61.8% (278)	2.8% (−7.3% to 12.8%)	0.591
Male	37.7% (211)	35.5% (39)	38.2% (172)	−2.8% (−12.8% to 7.3%)	-
**Race/Ethnicity, % (n)**					
White (non-hispanic)	94.3% (528)	96.4% (106)	93.8% (422)	2.6% (−6.7% to 1.6%)	0.295
Other	5.7% (32)	3.6% (4)	6.2% (28)	−2.6% (−1.6% to 6.7%)	-
**Primary Language, % (n)**					
English	96.6% (541)	97.3% (107)	96.4% (434)	−0.8% (−4.3% to 2.7%)	0.667
Other	3.4% (19)	2.7% (3)	3.6% (16)	0.8% (−2.7% to 4.3%)	-
**Insurance Type, % (n)**					
Commercial	3.2% (18)	5.4% (6)	2.7% (12)	2.8% (−1.7% to 7.3%)	0.069
Medicaid	1.3% (7)	0% (0)	1.6% (7)	−1.6% (−0.42% to 2.7%)	**0.021**
Medicare	94.5% (529)	91.8% (101)	95.1% (428)	−3.3% (−8.8 to 2.3%)	0.096
Other Governmental ^§^	8.9% (5)	2.7% (3)	0.4% (2)	2.3 (−0.8% to 5.4%)	**0.046**
Uninsured	0.2% (1)	-	0.2% (1)	−0.2% (−0.7% to 0.2%)	**<0.001**
**Employment Status, % (n)**					
Working	6.4% (36)	9.1% (10)	5.8% (26)	3.3% (−2.5% to 9.1%)	0.204
Retired or Inactive	93.6% (524)	90.9% (100)	94.2% (424)	−3.3 (−9.1% to 2.5%)	-
**Distance, Miles (SD)**					
Mean	14.0 (22.2)	12.6 (22.7)	14.3 (22.1)	-	0.458
**Estimated Household Income, $ (SD)**				-	
Mean (thousands)	111 (68)	112 (60)	110 (69)		0.740
**AMD Severity, % (n)**					
Intravitreal Injections ^‡^	90.5% (507)	88.2% (97)	91.1% (410)	−2.9% (−9.5% to 3.7%)	0.347
Observation	9.5% (53)	11.8% (13)	8.9% (40)	2.9% (3.7% to 9.5%)	-
Bilateral nAMD	48.0% (269)	60.9% (67)	45.1% (202)	16.0% (5.9% to 26.3%)	**0.003**
Unilateral nAMD	52.2% (291)	39.1% (43)	54.9% (248)	−16.0% (−26.3% to −5.9%)	**-**
**Visual Acuity, LogMAR (SD)**					
Better Eye Vision	0.368 (0.357)	0.514 (0.408)	0.332 (0.334)	-	**<0.001**
Worse Eye Vision	0.975 (0.829)	1.241 (0.910)	0.910 (0.796)	-	**<0.001**
**Visual Status, % (n)**					
Mild Visual Impairment	36.3% (203)	55.5% (61)	31.6% (142)	23.9% (13.6% to 34.2%)	**<0.001**
Low Vision	15.7% (88)	31.8% (35)	11.8% (53)	20.0% (10.8% to 29.3%)	**<0.001**
Legal Blindness	7.0% (39)	13.6% (15)	5.3% (24)	8.3% (15.1% to 1.5%)	**0.002**
Monocular	30.0% (168)	39.1% (43)	27.8% (125)	11.3% (21.4% to 1.3%)	**0.020**

^†^ Significance is marked in bold (*p* < 0.05). ^‡^ Includes those patients receiving injections as-needed (pro re nata). ^§^ Other governmental insurance included Tr-Service Healthcare Program, Federal Employee Health Benefits Program, and other federal and local government-sponsored health programs. nAMD = Neovascular age-related macular degeneration. LogMAR = Logarithm of the minimum angle of resolution. SD = Standard deviation.

**Table 2:** Multiple Logistic Regression Analysis of Variables Associated with Referral for Low Vision Evaluation.

Characteristic	β	Standard Error	Wald χ^2^	Odds Ratio	95% CI		*p* ^†^
					Lower Bound	Upper Bound	
**Visual Status**							
Low Vision	1.167	0.263	19.717	3.214	1.920	5.380	**<0.001**
**nAMD Severity**							
Bilateral Disease	0.465	0.229	4.139	1.592	1.017	2.492	**0.042**
**Health Insurance Type** **(Relative to Medicare)**							
Commercial	1.06	0.521	4.149	2.887	1.041	8.009	**0.042**
Medicaid ^‡^	−19.337	15,157	0	0	-	-	0.999
Other Governmental ^§^	1.776	0.985	3.25	5.905	0.857	40.718	0.071
Uninsured ^‡^	−20.428	40,192	0	0	-	-	1.0
**Constant**	−1.943	0.178	118.636	0.143	-	-	**<0.001**

^†^ Significance is marked in bold (*p* < 0.05). ^‡^ The logistic regression model could not compute meaningful coefficients for the effect of having Medicaid health insurance or being uninsured because of perfect prediction and small sample size in these categories. ^§^ Other governmental insurance included Tr-Service Healthcare Program, Federal Employee Health Benefits Program, and other federal and local government-sponsored health programs.

## Data Availability

The datasets generated and/or analyzed during the current study are not publicly available due to the use of confidential patient health record data. Participants of this study did not agree for their data to be shared publicly, so supporting data is not available.

## References

[1] Centers for Disease Control and Prevention (CDC) Prevalence of Age-Related Macular Degeneration (AMD). https://www.cdc.gov/vision-health-data/prevalence-estimates/amd-prevalence.html?CDC_AAref_Val=https://www.cdc.gov/visionhealth/vehss/estimates/amd-prevalence.html.

[2] Centers for Disease Control and Prevention (CDC) (2020). Fast Facts of Common Eye Disorders. https://www.cdc.gov/vision-health/data-research/vision-loss-facts/?CDC_AAref_Val=https://www.cdc.gov/visionhealth/basics/ced/fastfacts.htm.

[3] Khorrami-Nejad M., Sarabandi A., Akbari M.R., Askarizadeh F. (2016). The impact of visual impairment on quality of life. Med. Hypothesis Discov. Innov. Ophthalmol..

[4] Kempen G.I., Ballemans J., Ranchor A.V., van Rens G.H., Zijlstra G.A. (2012). The impact of low vision on activities of daily living, symptoms of depression, feelings of anxiety and social support in community-living older adults seeking vision rehabilitation services. Qual. Life Res..

[5] Fonteh C.N., Mathias M.T., Mandava N., Manoharan N., Lynch A.M., Navo R., Patnaik J.L., University of Colorado Retina Research Group (2022). Mental health and visual acuity in patients with age-related macular degeneration. BMC Ophthalmol..

[6] Ehrlich J.R., Ramke J., Macleod D., Burn H., Lee C.N., Zhang J.H., Waldock W., Swenor B.K., Gordon R., Congdon N. (2021). Association between vision impairment and mortality: A systematic review and meta-analysis. Lancet Glob. Health.

[7] Marques A.P., Ramke J., Cairns J., Butt T., Zhang J.H., Muirhead D., Jones I., Ah Ton B.A.M., Swenor B.K., Faal H. (2021). Global economic productivity losses from vision impairment and blindness. EClinicalMedicine.

[8] Chaudhuri M., Hassan Y., Bakka Vemana P.P.S., Bellary Pattanashetty M.S., Abdin Z.U., Siddiqui H.F. (2023). Age-Related Macular Degeneration: An Exponentially Emerging Imminent Threat of Visual Impairment and Irreversible Blindness. Cureus.

[9] Martin D.F., Maguire M.G., Fine S.L., Ying G.S., Jaffe G.J., Grunwald J.E., Toth C., Redford M., Ferris F.L., Comparison of Age-related Macular Degeneration Treatment Trials (CATT) Research Group (2012). Ranibizumab and bevacizumab for treatment of neovascular age-related macular degeneration: Two-years results. Ophthalmology.

[10] Elshout M., Webers C.A., van der Resi M.I., de Jong-Hesse Y., Schouten J.S. (2017). Tracing the natural course of visual acuity and quality of life in neovascular age-related macular degeneration: A systematic review and quality of life study. BMC Ophthalmol..

[11] Owsley C., McGwin G., Lee P.P., Wasserman N., Searcey K. (2009). Characteristics of low-vision rehabilitation services in the United States. Arch. Ophthalmol..

[12] Stelmack J.A., Tang X.C., Reda D.J., Rinne S., Mancil R.M., Massof R.W., LOVIT Study Group (2008). Outcomes of the Veterans Affairs Low Vision Intervention Trial (LOVIT). Arch. Ophthalmol..

[13] Stolwijk M.L., van Nispen R.M.A., van der Ham A.J., Veenman E., van Rens G.H.M.B. (2023). Barriers and facilitators in the referral pathways to low vision services from the perspective of patients and professionals: A qualitative study. BMC Health Serv. Res..

[14] Larochelle R.D., Patnaik J.L., Lynch A.M., Mandeva N., Hansn K., University of Colorado Retina Research Group (2022). Low vision referral patterns in intermediate age-related macular degeneration. Curr. Eye Res..

[15] American Academy of Ophthalmology (AOA) Initiative in Vision Rehabilitation. https://www.aao.org/education/low-vision-and-vision-rehab.

[16] World Health Organization (2019). World Report on Vision. https://iris.who.int/bitstream/handle/10665/328717/9789241516570-eng.pdf?sequence=18.

[17] Kuo K.H., Anjum S., Nguyen B., Marx J.L., Roh S., Ramsey D.J. (2021). Utilization of remote diabetic retinal screening in a suburban healthcare system. Clin. Ophthalmol..

[18] Internal Revenue Service (2021). SOI Tax Stats—Individual Income Tax Statistics—ZIP Code Data (SOI). https://www.irs.gov/statistics/soi-tax-stats-individual-income-tax-statistics-zip-code-data-soi.

[19] Stein J.D., Newman-Casey P.A., Kim D.D., Nwanyanwu K.H., Johnson M.W., Hutton D.W. (2013). Cost-effectiveness of various interventions for newly diagnosed diabetic macular edema. Ophthalmology.

[20] Centers for Medicare & Medicaid Services (CMS) Physician Fee Schedule. https://www.cms.gov/Medicare/Medicare-Fee-for-Service-Payment/PhysicianFeeSched.

[21] Kaleem M.A., West S.K., Im L., Swenor B.K. (2018). Referral to low vision services for glaucoma patients: Referral criteria and barriers. J. Glaucoma.

[22] Jackson M.L., Virgili G., Shepherd J.D., Di Nome M.A., Fletcher D.C., Kaleem M.A., Lam L.A., Lawrence L.M., Sunness J.S., Riddering A.T. (2023). Vision Rehabilitation Preferred Practice Pattern^®^. Ophthalmology.

[23] Guo X., Boland M.V., Swenor B.K., Goldstein J.E. (2023). Low vision rehabilitation service utilization before and after implementation of a clinical decision support system in ophthalmology. JAMA Netw. Open.

[24] Goldstein J.E., Guo X., Boland M.V., Swenor B.K. (2020). Low vision care—Out of site. Out of mind. Ophthalmic Epidemiol..

[25] Guo X., Swenor B.K., Smith K., Boland M.V., Goldstein J.E. (2021). Developing an ophthalmology clinical decision support system to identify patients for low vision rehabilitation. Transl. Vis. Sci. Technol..

[26] Nilsson U.L., Nilsson S.E. (1986). Rehabilitation of the visually handicapped with advanced macular degeneration. A follow-up study at the Low Vision Clinic, Department of Ophthalmology, University of Linköping. Doc. Ophthalmol..

[27] Parravano M., Petri D., Maurutto E., Lucenteforte E., Menchini F., Lanzetta P., Varano M., van Nispen R.M.A., Virgili G. (2021). Association between visual impairment and depression in patients attending eye clinics: A meta-analysis. JAMA Ophthalmol..

[28] Cahill M.T., Banks A.D., Stinnett S.S., Toth C.A. (2005). Vision-related quality of life in patients with bilateral severe age-related macular degeneration. Ophthalmology.

[29] Mangione C.M., Lee P.P., Gutierrez P.R., Spritzer K., Berry S., Hays R.D. (2001). National Eye Institute Visual Function Questionnaire Field Test Investigators. Development of the 25-item National Eye Institute Visual Function Questionnaire. Arch. Ophthalmol..

[30] Patnaik J.L., Lynch A.M., Pecen P.E., Jasso M., Hanson K., Mathias M.T., Palestine A.G., Mandava N. (2021). The impact of advanced age-related macular degeneration on the National Eye Institute’s Visual Function Questionnaire-25. Acta Ophthalmol..

[31] Binns A.M., Bunce C., Dickinson C., Harper R., Tudor-Edwards R., Woodhouse M., Linck P., Suttie A., Jackson J., Lindsay J. (2012). How effective is low vision service provision? A systematic review. Surv. Ophthalmol..

[32] Scott I.U., Smiddy W.E., Schiffman J., Feuer W.J., Pappas C.J. (1999). Quality of life of low-vision patients and the impact of low-vision services. Am. J. Ophthalmol..

[33] Browne C., Brazier J., Carlton J., Alavi Y., Jofre-Bonet M. (2012). Estimating quality-adjusted life years from patient-reported visual functioning. Eye.

[34] Center for Medicare and Medicaid Services ASP Pricing Files. https://www.cms.gov/medicare/payment/part-b-drugs/asp-pricing-files.

[35] Kiss S., Malangone-Monaco E., Wilson K., Varker H., Stetsovsky D., Smith D., Garmo V. (2020). Real-world injection frequency and cost of ranibizumab and aflibercept for the treatment of neovascular age-related macular degeneration and diabetic macular edema. J. Manag. Care Spec. Pharm..

[36] Williams A.M., Sahel J.A. (2022). Addressing Social Determinants of Vision Health. Ophthalmol. Ther..

